# The LIM domain protein nTRIP6 acts as a co-repressor for the transcription factor MEF2C in myoblasts

**DOI:** 10.1038/srep27746

**Published:** 2016-06-13

**Authors:** Denise Kemler, Oliver Dahley, Sven Roßwag, Margarethe Litfin, Olivier Kassel

**Affiliations:** 1 Karlsruhe Institute of Technology (KIT), Institute of Toxicology and Genetics, Karlsruhe, Germany

## Abstract

The transcription factor Myocyte enhancer factor 2C (MEF2C) plays a key role in the late differentiation of skeletal muscle progenitor cells, the so-called myoblasts. During myoblast differentiation, both MEF2C expression and transcriptional activity are regulated. We have reported that nTRIP6, the nuclear isoform of the focal adhesion LIM domain protein TRIP6, acts as an adaptor transcriptional co-activator for several transcription factors. It interacts with the promoter-bound transcription factors and consequently mediates the recruitment of other co-activators. Based on a described interaction between MEF2C and TRIP6 in a yeast-two-hybrid screen, we hypothesised a co-regulatory function of nTRIP6 for MEF2C. In proliferating myoblasts, nTRIP6 interacted with MEF2C and was recruited together with MEF2C to the MEF2-binding regions of the MEF2C target genes *Myom2*, *Mb*, *Tnni2* and *Des*. Silencing nTRIP6 or preventing its interaction with MEF2C increased MEF2C transcriptional activity and increased the expression of these MEF2C target genes. Thus, nTRIP6 acts as a co-repressor for MEF2C. Mechanistically, nTRIP6 mediated the recruitment of the class IIa histone deacetylase HDAC5 to the MEF2C-bound promoters. In conclusion, our results unravel a transcriptional co-repressor function for nTRIP6. This adaptor co-regulator can thus exert either co-activator or co-repressor functions, depending on the transcription factor it interacts with.

The Myocyte enhancer factor 2 (MEF2) family of MADS (MCM1, Agamous, Deficiens and SRF) transcription factors, which comprises four members in vertebrates (MEF2A-D), are expressed in numerous tissues and organs, where they control differentiation programmes^1^. MEF2s are highly expressed in skeletal muscle, where they play a key role in terminal differentiation and maturation^2^,^3^,^4^. These factors are not able to activate the myogenic programme on their own, but they synergize with other myogenic determinants such as the transcription factor MYOD to promote myofibre differentiation^5^. Amongst MEF2 proteins, MEF2C plays a unique role in the late phases of myogenesis. In particular, it is essential for myofibre maturation by promoting the expression of late differentiation genes such as sarcomeric proteins^6^,^7^.

Although required at late stages of differentiation, MEF2C is already expressed in proliferating myoblasts^8^,^9^. Thus, its transcriptional activity has to be tightly controlled during myoblast proliferation and differentiation. Several mechanisms have been proposed that repress the myogenic activity of MEF2C in proliferating myoblasts. In particular, one of these mechanisms involves a dynamic regulation of the interaction between MEF2C and the transcriptional co-repressors class IIa histone deacetylases (HDACs) such as HDAC4 and −5: in proliferating myoblasts MEF2C interacts with HDACs and its activity is repressed, and myogenic signals disrupt this interaction, thereby alleviating the repression of MEF2C activity^10^,^11^,^12^,^13^,^14^. Similarly, several transcriptional co-activators such as GRIP-1 and CARM1 have been shown to dynamically activate MEF2C activity during myogenesis^15^,^16^.

A search of the IntAct database^17^ for MEF2C-interacting proteins that could modulate its activity revealed TRIP6 as a candidate. TRIP6 belongs to the ZYXIN family of LIM domain-containing adaptor proteins^18^,^19^. The LIM domain, first identified in the Lin-11, Isl-1 and Mec-3 homeodomain transcription factors, is defined as a cysteine-rich motif organised as a double zinc finger structure, which mediates protein-protein interactions^20^,^21^. TRIP6, like the other members of the ZYXIN family, carries three C-terminal LIM domains. Furthermore, a nuclear export signal (NES) within the N-terminal pre-LIM region is responsible for the cytoplasmic localisation of TRIP6^22^. TRIP6 is enriched at sites of focal adhesion and cell-cell contacts and is involved in adhesion and migration^23^,^24^,^25^. Surprisingly however, members of this so-called focal adhesion LIM domain protein family, including TRIP6, also function as transcriptional co-regulators in the nucleus^18^,^19^,^26^. We have previously demonstrated that the nuclear functions of TRIP6 are mediated by a shorter isoform exclusively expressed in the nucleus, and therefore termed nTRIP6^27^. Here, nTRIP6 acts as a transcriptional co-activator for the transcription factors AP-1, NF-κB and the glucocorticoid receptor (GR)^27^,^28^,^29^. nTRIP6 interacts with these transcription factors via its LIM domains, is recruited to the transcription factor-bound target promoters via this interaction, and is required for the activation of transcription. We have recently shown that nTRIP6 exerts this co-activator function by acting as an adaptor protein, mediating the recruitment of the Mediator complex subunit THRAP3 to the transcription factor-bound promoter^30^.

Given the putative interaction between nTRIP6 and MEF2C, we hypothesised that nTRIP6 could act as a co-activator for MEF2C. We report here that surprisingly, nTRIP6 acts as a co-repressor for MEF2C in myoblasts. nTRIP6 interacts via its N-terminal pre-LIM region with MEF2C in the nucleus of myoblasts, is recruited together with MEF2C to the regulatory regions of MEF2C target genes and represses their expression. Mechanistically, nTRIP6 interacts with HDAC5 and mediates its recruitment to the MEF2C-bound regulatory region. Thus, our data document a novel, unexpected adaptor function for nTRIP6 in the regulation of a transcription factor involved in myogenesis.

## Results

### MEF2C interacts with the N-terminal pre-LIM region of nTRIP6

**Figure 1:**
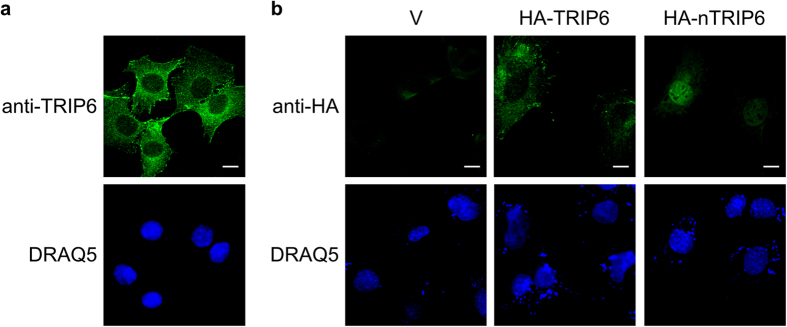
Subcellular localisation of TRIP6 and nTRIP6 in myoblasts. (**a**) C2C12 cells were subjected to immunofluorescence analysis using an antibody recognising both TRIP6 and nTRIP6. Nuclei were counterstained using DRAQ5. (**b**) C2C12 cells were transfected with an empty vector (V) or an expression vector for either HA-tagged TRIP6 or nTRIP6, subjected to immunofluorescence analysis using an anti-HA antibody and counterstained using DRAQ5. (**a**,**b**) Representative confocal micrographs are shown, scale bars: 10 μm.

**Figure 2:**
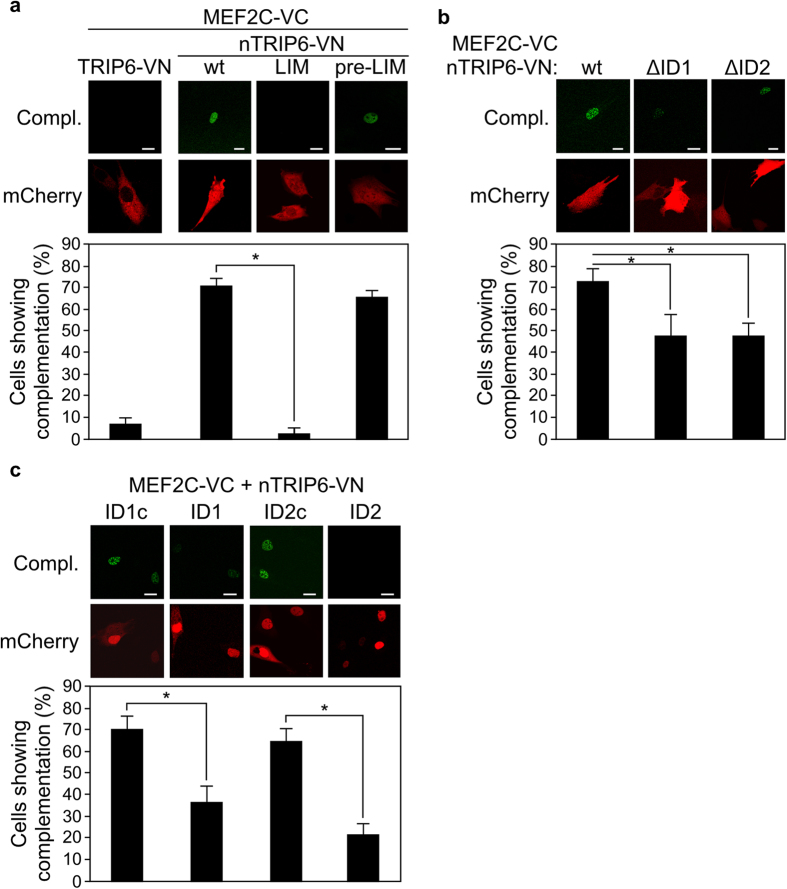
nTRIP6 interacts with Mef2C via its N-terminal pre-LIM region. (**a**) C2C12 cells were co-transfected with expression vectors for MEF2C fused to the C-terminal half of Venus (VC) and for either TRIP6, nTRIP6 (wt), nTRIP6 LIM domains only (LIM) or nTRIP6 lacking the LIM domains (pre-LIM) fused to the N-terminal half of Venus (VN), together with an expression vector for mCherry as a transfection control. (**b**) C2C12 cells were co-transfected with MEF2C-VC and either nTRIP6 (wt), nTRIP6 lacking the interaction domain 1 (ΔID1) or nTRIP6 lacking the interaction domain 2 (ΔID2) fused to VN, together with mCherry. (**c**) C2C12 cells were co-transfected with MEF2C-VC and nTRIP6-VN, together with a peptide corresponding to the sequence of the interaction domain 1 fused to a nuclear localisation signal (NLS) and to mCherry (ID1), a scrambled version of the ID1 peptide (ID1c), a peptide corresponding to the sequence of the interaction domain 2 fused to an NLS and to mCherry (ID2), or a scrambled version of the ID2 peptide (ID2c). (**a**–**c**) Venus complementation (Compl.) was imaged by confocal microscopy and representative cells are shown (Scale bar: 10 μm). The number of cells showing Venus complementation is presented as percentage of transfected, mCherry positive cells (mean ± SD of three independent experiments; *P < 0.05).

Given the reported interaction between MEF2C and TRIP6, and our previous results showing that the short nuclear isoform nTRIP6 is responsible for the transcriptional co-regulatory function of TRIP6, we first studied the subcellular localisation of TRIP6 and nTRIP6 in C2C12 myoblasts. Immunoreactivity to an antibody recognising both isoforms revealed a predominantly cytosolic staining with an enrichment at focal adhesions and cell-cell contacts, as expected, as well as a weaker staining in the nucleus (Fig. 1a). However, ectopically expressed HA-tagged TRIP6 was only detected in the cytosol, whereas HA-tagged nTRIP6 was exclusively nuclear (Fig. 1b). Thus, if one of the isoforms acts a transcriptional co-regulator for MEF2C in the nucleus, it has to be nTRIP6. We therefore investigated a putative function of nTRIP6 in regulating MEF2C transcriptional activity. As a first step, we studied whether the two proteins interacted in C2C12 myoblasts using bimolecular fluorescence complementation (BiFC) of the Venus fluorescent protein^31^. Venus complementation was detected in only 7.3 ± 2.2% of the cells co-transfected with MEF2C fused to the C-terminal half of Venus (VC) and the cytosolic isoform TRIP6 fused to the N-terminal half of Venus (VN), although the cells were efficiently transfected as indicated by their expression of the fluorescent protein mCherry used as a transfection control (Fig. 2a). However, Venus complementation was observed in the nucleus of 70.6 ± 3.4% of the cells co-transfected with MEF2C-VC and nTRIP6, the nuclear isoform, fused to VN (Fig. 2a), confirming that the two proteins indeed interact in the nucleus of myoblasts. Furthermore, Venus complementation was observed in the nucleus of 65.5 ± 3.2% of the cells co-transfected with MEF2C-VC and an nTRIP6 construct lacking the LIM domains (pre-LIM) fused to VN. However, complementation was detected in only 2.1 ± 3.0% of the cells co-transfected with MEF2C-VC and the LIM domains alone fused to VN (Fig. 2a). Importantly fusion to VC or VN did not affect the nuclear localisation of the fused proteins, i.e. TRIP6-VN was exclusively cytosolic whereas MEF2C-VC and the nTRIP6 constructs fused to VN were exclusively nuclear (Supplementary Fig. S1a). Furthermore, the nTRIP6 constructs were expressed at comparable levels (Supplementary Fig. S1b). These results show that, surprisingly, MEF2C interacts with the N-terminal pre-LIM region and not with the LIM domains of nTRIP6, as it is the case for the known transcription factors co-activated by nTRIP6^27^,^28^,^29^. We have recently described two interaction domains (ID) within the N-terminal pre-LIM region of nTRIP6, ID1 corresponding to amino acid positions 175–187 and ID2 to positions 253–265^30^. We therefore tested whether these domains were involved in the interaction with MEF2C. Both the deletion of ID1 and of ID2 significantly reduced the number of cells showing Venus complementation in the BiFC assay (Fig. 2b), although both constructs were expressed in the nucleus (Supplementary Fig. S1a) and at similar levels as the wild type nTRIP6 construct (Supplementary Fig. S1c). Furthermore, two genetically encoded peptides, corresponding to ID1 and ID2 fused to mCherry and to a nuclear localisation signal (NLS), both significantly reduced the number of cells showing complementation between nTRIP6-VN and MEF2C-VC, as compared to ID1c and ID2c, the control, scrambled versions of the peptides (Fig. 2c). These results suggest that both domains participate in the interaction between nTRIP6 and MEF2C. Together, these results show that nTRIP6 interacts with MEF2C in the nucleus of myoblasts via discrete domains of its N-terminal pre-LIM region.

### nTRIP6 acts as a co-repressor for MEF2C

**Figure 3:**
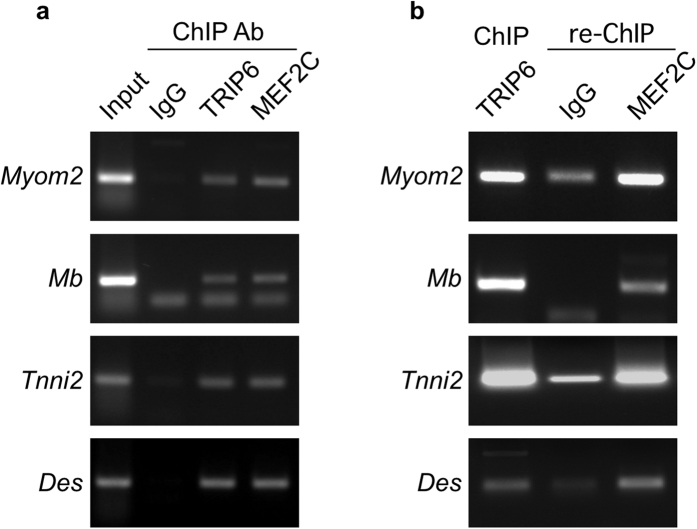
nTRIP6 and MEF2C co-occupy MEF2C-dependent promoters. (**a**) Chromatin immunoprecipitation (ChIP) was performed in C2C12 cells using the indicated antibodies (Ab). (**b**) Chromatin immunoprecipitated with the anti-TRIP6 antibody was eluted and subjected to a re-ChIP using either the anti-MEF2C or the isotype control Ab. (**a**,**b**) PCR was performed with primers flanking the MEF2 binding regions of the indicted genes. Representative gels are shown.

If nTRIP6 co-regulates MEF2C, it should be recruited to the MEF2-binding regions of MEF2C target genes. To address this question we performed chromatin immunoprecipitation (ChIP) experiments in C2C12 cells. Indeed, nTRIP6 was recruited to MEF2C-binding regions of the MEF2C target genes *Myomesin 2* (*Myom2*)^7^, *Myoglobin* (*Mb*)^32^, *Troponin I type 2* (*Tnni2*)^33^ and *Desmin* (*Des*)^34^,^35^ (Fig. 3a). To confirm the specificity of our anti-TRIP6 antibody, we performed the same ChIP in cells transfected with an siRNA targeting *Trip6* mRNA and down-regulating the expression of both TRIP6 and nTRIP6 (Supplementary Fig. S2a)^28^. Transfection with the siRNA significantly reduced the recruitment of nTRIP6 to the MEF2C binding sites of the *Myom2, Mb, Tnni2* and *Des* genes, as compared to a control siRNA (Supplementary Fig. S2b). Furthermore, re-ChIP assays confirmed that MEF2C and nTRIP6 co-occupied the MEF2 binding sites of these genes (Fig. 3b).

**Figure 4:**
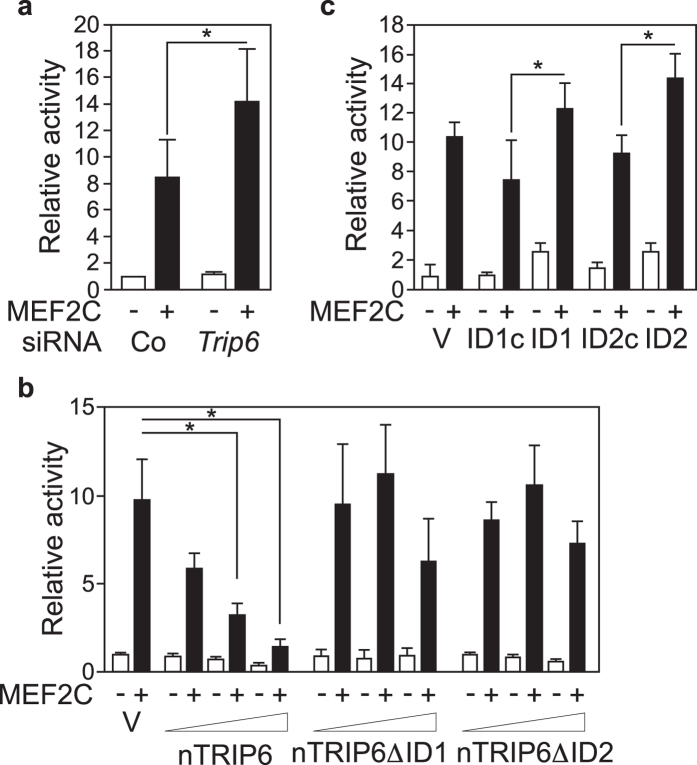
nTRIP6 represses MEF2C transcriptional activity. (**a**) C2C12 cells were transfected with either an siRNA targeting *Trip6* mRNA or a control siRNA (Co), and 24 h later with a MEF2-dependent reporter gene together with an expression vector for β-galactosidase, and with either an expression vector for MEF2C (+) or an empty vector (−). Normalised luciferase activities are plotted relative to the control siRNA, empty vector transfected cells (mean ± SD of three independent experiments; *P < 0.05). (**b**) C2C12 cells were co-transfected with the MEF2-dependent reporter gene, the expression vector for β-galactosidase, either the expression vector for MEF2C (+) or the empty vector (−), together with either a control empty vector (V) or increasing amounts of an expression vector for nTRIP6, for nTRIP6 lacking the interaction domain 1 (nTRIP6ΔID1) or nTRIP6 lacking the interaction domain 2 (nTRIP6ΔID2). (**c**) C2C12 cells were co-transfected with the MEF2-dependent reporter gene, the expression vector for β-galactosidase, either the expression vector for MEF2C (+) or the empty vector (−), together with either a control empty vector (V), the mCherry-NLS fusion of either the ID1 peptide, its scrambled version (ID1c), the ID2 peptide or its scrambled version (ID2c). (**b**,**c**) Normalised luciferase activities are plotted relative to the empty vector control (mean ± SD of three independent experiments; *P < 0.05).

Thus, nTRIP6 interacts with MEF2C and is recruited together with MEF2C to the regulatory regions of MEF2C target genes. Therefore, nTRIP6 might act as a co-activator for MEF2C. If so, it should positively regulate MEF2C transcriptional activity. However, in a reporter gene assay, transfection of the siRNA targeting *Trip6* mRNA increased the transcriptional activity of co-transfected MEF2C (Fig. 4a and Supplementary Fig. S3a). Conversely, over-expression of nTRIP6 dose-dependently decreased the MEF2C-induced expression of the reporter gene (Fig. 4b). Control Western Blots showed that this inhibition was not due to a lower expression of MEF2C (Supplementary Fig. S3b). This repressive effect was not observed upon over-expression of nTRIP6 lacking either ID1 or ID2 (Fig. 4b), suggesting that it is mediated by the interaction between MEF2C and the N-terminal pre-LIM region of nTRIP6. To confirm this finding, we made use of the peptides blocking the interaction between MEF2C and nTRIP6. Transfection of either the ID1 or the ID2 peptide increased MEF2C transcriptional activity in the reporter gene assay, while the control peptides had no effect (Fig. 4c). Control western blots for these reporter gene assays are presented in Supplementary Fig. S3.

Together, our results show that nTRIP6 interacts with MEF2C, is recruited together with MEF2C to the regulatory regions of MEF2C target genes and represses MEF2C transcriptional activity. Thus, nTRIP6 is a transcriptional co-repressor for MEF2C.

### nTRIP6 mediates the recruitment of HDAC5 to MEF2C target gene promoters

**Figure 5:**
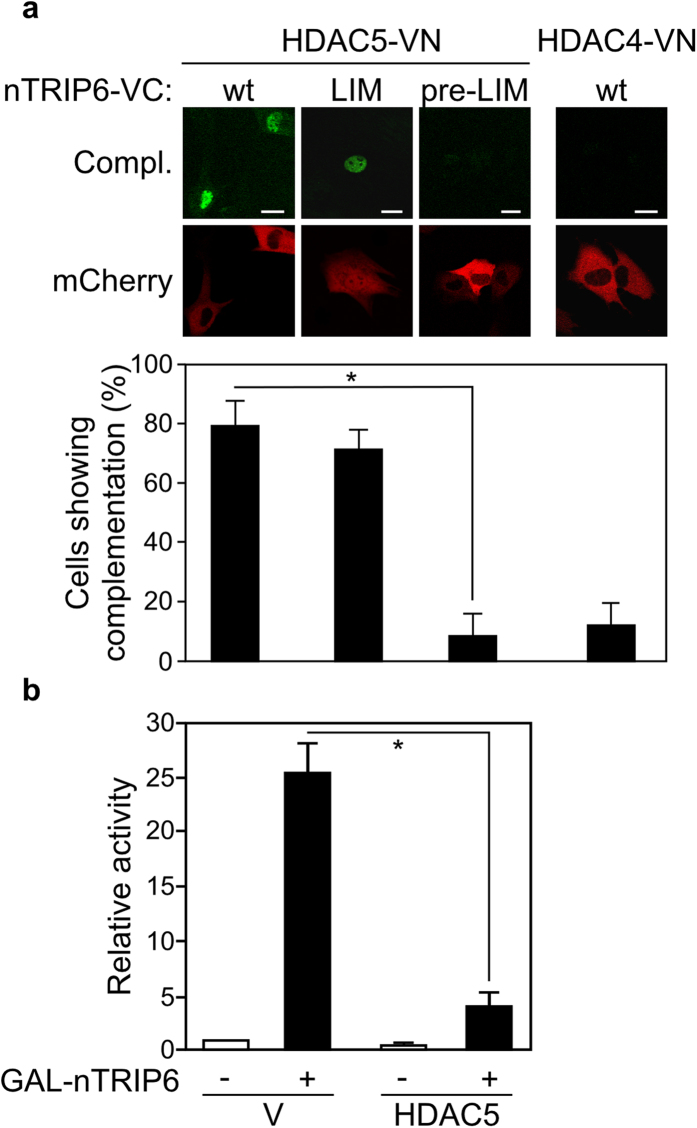
nTRIP6 interacts with HDAC5. (**a**) C2C12 cells were co-transfected with expression vectors for either HDAC5 or HDAC4 fused to the N-terminal half of Venus (VN) and for either nTRIP6 (wt), nTRIP6 LIM domains only (LIM) or nTRIP6 lacking the LIM domains (pre-LIM) fused to the C-terminal half of Venus (VC), together with an expression vector for mCherry as a transfection control. Venus complementation (Compl.) was imaged by confocal microscopy and representative cells are shown (top, scale bar: 20 μm). The number of cells showing Venus complementation is presented as percentage of transfected, mCherry positive cells (bottom, mean ± SD of three independent experiments; *P < 0.05). (**b**) C2C12 cells were co-transfected with a GAL4-responsive reporter gene together with an expression vector for β-galactosidase, with either an expression vector for nTRIP6 fused to the GAL4 DNA binding domain (GAL-nTRIP6; +) or an empty vector (−), and with either an expression vector for HDAC5 or an empty vector (V). Normalised luciferase activities are plotted relative to the empty vector control (mean ± SD of three independent experiments; *P < 0.05).

nTRIP6 does not harbour any functional domain known from other classical co-repressors. Our current understanding of nTRIP6 function as a co-activator for other transcription factors is that it acts as a bridging factor, recruiting through its LIM-domains other co-activators such as THRAP3 to target promoters^30^. A logical hypothesis is therefore that nTRIP6 recruits other co-repressors to MEF2C-bound target genes. Given that MEF2C is repressed by class IIa histone deacetylases (HDACs) such as HDAC4 and 5^10^,^11^,^12^,^13^,^14^, we hypothesised that nTRIP6 represses MEF2C by mediating the recruitment of class IIa HDACs. We first tested whether nTRIP6 interacted with HDAC5 in the nucleus of C2C12 myoblasts using the BiFC assay. Venus complementation was observed in the nucleus of 79.5 ± 8.2 cells co-transfected with nTRIP6 fused to VC and HDAC5 fused to VN (Fig. 5a). HDAC5 did not interact with the N-terminal pre-LIM region, but with the LIM domains of nTRIP6 (Fig. 5a). Furthermore, Venus complementation was detected in only 12.0 ± 7.6% of the cells co-transfected with nTRIP6 fused to VC and HDAC4 fused to VN (Fig. 5a), illustrating the specificity of the interaction between nTRIP6 and HDAC5. Immunofluorescence analysis confirmed the proper expression and localisation of HDAC4-VN and HDAC5-VN (Supplementary Fig. S4a). We then verified that the interaction between nTRIP6 and HDAC5 was associated with transcriptional repression. We made use of our previous observation that a fusion between nTRIP6 and the DNA-binding domain of the yeast transcription factor GAL4 (GAL_DBD_-LIM) acts as a pseudo-transcription factor^30^, driving the expression of a luciferase reporter gene under the control of the GAL4 upstream activating sequence (GAL-UAS). In C2C12 cells GAL_DBD_-nTRIP6 activated the reporter gene transcription, as compared to the GAL_DBD_ alone (Fig. 5b). This transcriptional activity was repressed by co-transfection of HDAC5 (Fig. 5b). This result confirms that the interaction between nTRIP6 and HDAC5 results in transcriptional repression.

**Figure 6:**
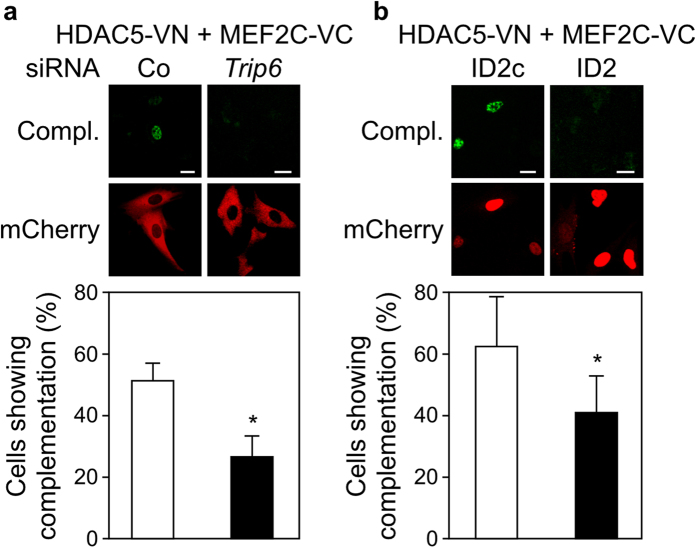
nTRIP6 mediates the interaction between MEF2C and HDAC5. (**a**) C2C12 cells were transfected with either an siRNA targeting *Trip6* mRNA or a control siRNA (Co), and 24h later with expression vectors for HDAC5 fused to the N-terminal half of Venus (VN) and MEF2C fused to the C-terminal half of Venus (VC), together with an expression vector for mCherry as a transfection control. (**b**) C2C12 cells were co-transfected with expression vectors for HDAC5-VN and MEF2C-VC together with either the mCherry-NLS fusion of the ID2 peptide or its scrambled version (ID2c). (**a**,**b**) Venus complementation (Compl.) was imaged by confocal microscopy and representative cells are shown (top, scale bar: 20 μm). The number of cells showing Venus complementation is presented as percentage of transfected, mCherry positive cells (bottom, mean ± SD of three independent experiments; *P < 0.05).

Given that nTRIP6 interacts with MEF2C via its pre-LIM region and with HDAC5 via its LIM domains, we hypothesised that it might serve as a bridging factor between MEF2C and HDAC5. To address this question, we studied the interaction between MEF2C and HDAC5 in a BiFC assay. MEF2C-VC and HDAC5-VN interacted in the nucleus of 50 to 60% of the transfected cells (Fig. 6a). A significant decrease in the interaction between MEF2C-VC and HDAC5-VN was observed in cells transfected with the *Trip6* siRNA, as compared to the control siRNA (Fig. 6a and Supplementary Fig. S4b). Furthermore, preventing the interaction between MEF2C and nTRIP6 using the ID2 blocking peptide inhibited the interaction between MEF2C and HDAC5 (Fig. 6b and Supplementary Fig. S4c).

**Figure 7:**
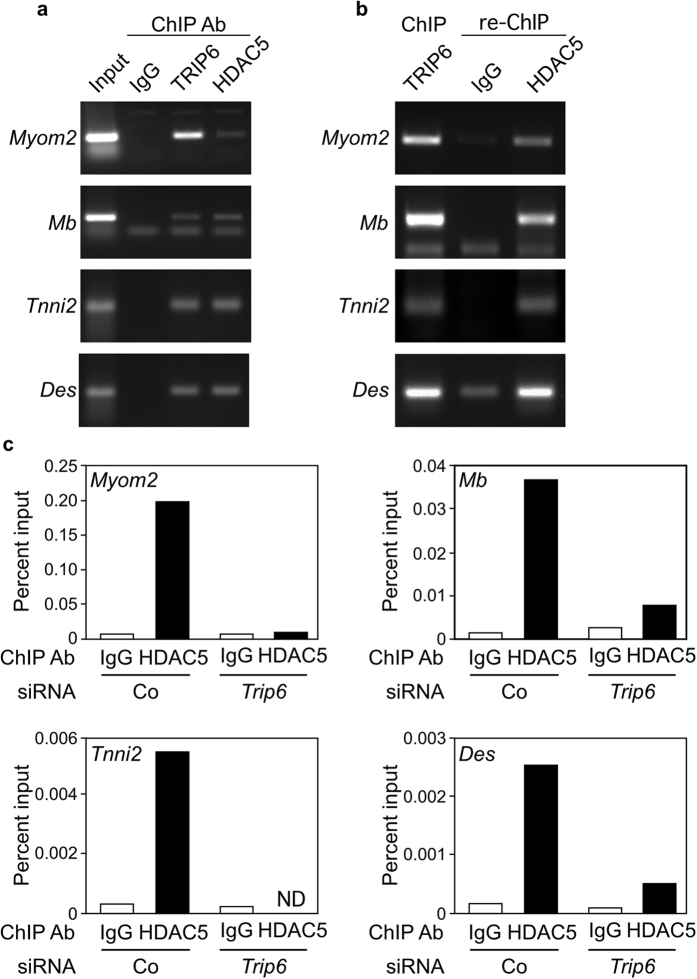
nTRIP6 mediates the recruitment of HDAC5 to MEF2C target promoters. (**a**) Chromatin immunoprecipitation (ChIP) was performed in C2C12 cells using the indicated antibodies (Ab). (**b**) Chromatin immunoprecipitated with the anti-TRIP6 antibody was eluted and subjected to a re-ChIP using either the anti-HDAC5 or the isotype control Ab. (**a**,**b**) PCR was performed with primers flanking the MEF2 binding regions of the indicted genes. Representative gels are shown. (**c**) C2C12 cells were transfected with either an siRNA targeting *Trip6* mRNA or a control siRNA (Co) and ChIP was performed using either an anti-HDAC5 or an isotype control Ab. Enrichments of the MEF2 binding regions of the indicted genes were determined by real-time PCR and are plotted as percent input. A representative experiment is shown; repetitions are presented in Supplementary Fig. S5.

These results show that nTRIP6 is required for the interaction between MEF2C and HDAC5. Thus, nTRIP6 might be required for the recruitment of HDAC5 to the regulatory regions of MEF2C target genes. In proliferating C2C12 myoblasts nTRIP6 and HDAC5 were both recruited to the MEF2-binding region of the *Myom2*, *Mb*, *Tnni2* and *Des* genes (Fig. 7a). Re-ChIP experiments confirmed that the two proteins co-occupied these regions (Fig. 7b). Finally, the recruitment of HDAC5 to the MEF2-binding region of these genes was strongly reduced in cells transfected with the *Trip6* siRNA (Fig. 7c and Supplementary Fig. S5). Thus, nTRIP6 mediates the recruitment of HDAC5 to the MEF2 binding sites of MEF2C target genes.

### nTRIP6 inhibits the expression of MEF2 target genes in myoblasts

**Figure 8:**
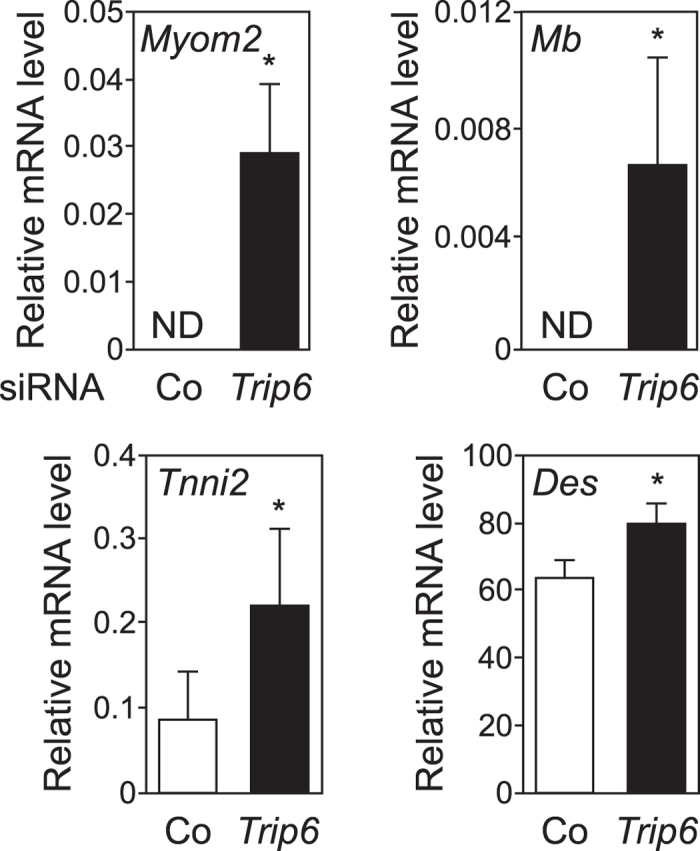
nTRIP6 represses the expression of MEF2C target genes in myoblasts. C2C12 cells were transfected with either an siRNA targeting *Trip6* mRNA or a control (Co) siRNA and the relative levels of the indicated mRNAs were determined by reverse transcription and real-time PCR. Results are plotted relative to the expression of the *Rplp0* gene (mean ± SD of three independent experiments; *P < 0.05).

To investigate the relevance of our findings, we studied the expression of MEF2 target genes in C2C12 myoblasts. Transfection of the *Trip6* siRNA significantly increased the mRNA level of *Myom2*, *Mb*, *Tnni2* and *Des*, as compared to the control siRNA (Fig. 8a). These results confirm that nTRIP6 represses the expression of MEF2C target genes in proliferating myoblast.

## Discussion

We report here a novel transcriptional co-repressor function of nTRIP6, the nuclear isoform of the LIM domain protein TRIP6, for the transcription factor MEF2C in proliferating myoblasts. Transcriptional co-repressor can be defined as factors which do not directly bind DNA but are recruited to the regulatory regions of target genes through a direct or indirect interaction with DNA-binding transcription factors, and which participate in the repression of transcription. Our data show that nTRIP6 interacts with MEF2C via its N-terminal pre-LIM region, is recruited together with MEF2C to the regulatory regions of MEF2C target genes, and represses the transcriptional activity of MEF2C. Thus, nTRIP6 is a MEF2C co-repressor. Of note, although the interaction domains of nTRIP6 pre-LIM region involved in the interaction with MEF2C are also present in TRIP6, we did not observe any interaction between TRIP6 and MEF2C. This most likely stems from the segregation of the two proteins in separate subcellular compartments, MEF2C being exclusively nuclear and TRIP6 exclusively cytosolic. This result confirms that also in myoblast, the nuclear functions of TRIP6 are mediated by the nuclear isoform nTRIP6.

Mechanistically, we show that nTRIP6 interacts via its LIM domains with the HDAC5 co-repressor, and mediates its recruitment to the regulatory regions of MEF2C target genes. nTRIP6 did not interact with HDAC4, another class IIa HDAC also involved in the repression of MEF2C activity^10^,^11^,^12^,^13^,^14^. This observation suggests the existence of different mechanisms regulating HDAC4 and HDAC5 function, and thus MEF2C activity in myoblasts. Such a difference has already been observed in another context: in fast type muscle fibres, low frequency electrical simulation promotes the nuclear export of HDAC4, but not of HDAC5^36^. Our observation of a selective interaction of nTRIP6 with HDAC5 further illustrates the complexity of MEF2C regulation by class IIa HDACs.

By mediating the recruitment of HDAC5 to MEF2C target genes, nTRIP6 acts as an adaptor co-repressor. In a similar manner, we have recently shown that when nTRIP6 co-activates other transcription factors such as AP-1, NF-κB and GR^27^,^28^,^29^, it acts as an adaptor co-activator, mediating the recruitment of the co-activator THRAP3^30^. Thus, it appears that nTRIP6 acts either as an adaptor co-activator or co-repressor, depending on the transcription factor it interacts with. Other members of the family of focal adhesion LIM domain protein have been shown to act both as co-activators and as co-repressors. For example, HIC-5 acts as a co-activator for the androgen receptor^37^,^38^ and for the GR^39^,^40^,^41^, and as a co-repressor for SMAD3^42^ and the LEF/TCF transcription factors^43^. Similarly, AJUBA, which acts as a co-repressor for the transcriptional repressors SNAIL/SLUG^44^,^45^,^46^ and GFI1^47^, was recently shown to act as a co-activator for PPARγ^48^ and LXR^49^. How can the very same adaptor co-regulator mediate the recruitment of either a co-activator or a co-repressor? Both for AP-1 co-activation and MEF2C co-repression, nTRIP6 uses its LIM domains to interact with and recruit the THRAP3 co-activator^30^ or the HDAC5 co-repressor (this study). One major difference lies in the way by which nTRIP6 is recruited to the transcription factor: nTRIP6 interacts with AP-1 via its LIM domains^27^, whereas it uses its pre-LIM region to interact with MEF2C. As stated above, AJUBA also acts as either a co-activator or a co-repressor, depending on the transcription factor it interacts with. Interestingly, AJUBA interacts with SNAIL via its LIM domains^46^ and mediates the recruitment of the PRMT5 co-repressor via its pre-LIM region^45^, whereas it interacts with PPARγ via its pre-LIM region and mediates the recruitment the p300/CBP co-activator via its LIM domains^48^. It is therefore tempting to speculate that the interaction with the transcription factors somehow affects the ability of the LIM domain protein to recruit either a co-activator or a co-repressor.

An alternative hypothesis would be that nTRIP6 recruits either co-activators or co-repressor depending on their availability or level in the nucleus. Indeed, the extent of MEF2C repression by HDAC5 depends on the amount of HDAC5 present in the nucleus^11^,^13^,^14^. These levels are high in proliferating myoblasts, and HDAC5 represses MEF2C activity before being exported to the cytosol upon differentiation^13^. Therefore, the ability of nTRIP6 to act as a co-repressor for MEF2C most likely depends on HDAC5 availability in the nucleus. Strengthening this hypothesis is the involvement of nTRIP6 in the repressive crosstalk between AP-1 by GR, a phenomenon referred to as trans-repression^50^,^51^,^52^,^53^. Without glucocorticoid treatment, nTRIP6 mediates the recruitment of the co-activator THRAP3 to the AP1-bound promoter. In the presence of glucocorticoids, GR is activated and translocates to the nucleus, interacts with nTRIP6 at the AP-1-bound promoter, displaces THRAP3 and represses AP-1 transcriptional activity^27^,^28^,^30^, in a way acting as an nTRIP6-dependent co-repressor. Both these examples suggest that indeed the ability of nTRIP6 to act either as a co-activator or a co-repressor depends on the availability of other co-regulators in the nucleus.

In myoblasts, MEF2C drives the expression of genes involved in late differentiation and in the maturation of muscle fibres^6^,^7^. However, MEF2C is already expressed in proliferating myoblasts^8^,^9^. Thus, its activity must be repressed to prevent premature differentiation, which is a least in part exerted by HDAC5^12^,^13^,^14^. Our results showing that in proliferating myoblasts nTRIP6 co-represses MEF2C suggest that nTRIP6 might participate in the inhibition of differentiation. The hypothesis of a role for nTRIP6 in the inhibition of differentiation in proliferating myoblasts is in part corroborated by our observation that silencing nTRIP6 promotes a strong increase in the expression of late differentiation genes, including sarcomeric proteins genes such as *Tnni2* and *Myom2*. Interestingly, AP-1 and NF-κB, which are co-activated by nTRIP6, have both been reported to repress the differentiation of proliferating myoblasts^54^,^55^,^56^. It is therefore tempting to speculate that nTRIP6 coordinates the inhibition of differentiation in proliferating myoblasts, by acting as a co-activator for “anti-differentiation” transcription factors and at the same time as a co-repressor for a pro-differentiation transcription factor.

## Materials and Methods

### Plasmid constructs

The MEF2C expression vector pCDNA.3-MEF2C and the MEF2-responsive reporter construct pGL3-TATA-Desmef3 were generous gifts from Eric N. Olson (University of Texas Southwestern Medical Center at Dallas, TX). The GAL4-UAS reporter construct GAL-Luc has been described^57^. pcDNA3.1-HDAC5-Flag (Addgene plasmid #13822) and pcDNA3.1-HDAC4-Flag (Addgene plasmid #13821) were gifts from Eric Verdin (University of California, San Francisco, CA)^58^. For bimolecular fluorescence complementation assays (BiFC)^31^,^59^, nTRIP6 and nTRIP6 lacking either the interaction domain 1 (nTRIP6ΔID1) or the interaction domain 2 (nTRIP6ΔID2) fused to the N-terminal half of the Venus fluorescent protein (VN), as well as nTRIP6, nTRIP6 pre-LIM region and nTRIP6 LIM domains fused to C-terminal half of the Venus fluorescent protein (VC) have been described^30^. TRIP6, nTRIP6 pre-LIM region and nTRIP6 LIM domains fused to VN were obtained by cloning the corresponding PCR fragments into pcDNA3.1-HA-VN^30^. To generate MEF2C fused to VC its coding sequence, without the stop codon, was PCR amplified using pCDNA.3-MEF2C as a template and subcloned into pcDNA3.1-HA-VC . HDAC5-Flag and HDAC4-Flag fused to VN were generated using the In-Fusion HD cloning kit (Clontech) according to the manufacturer’s instructions, and cloned into pcDNA3.1. pcDNA3.1-HA-nTRIP6, pcDNA-Gal_DBD_-nTRIP6 and pcDNA3.1-mCherry-NES have been described^27^,^29^,^30^. pcDNA3.1-HA-TRIP6 was generated by PCR amplification and subcloning into pcDNA3.1-HA^27^. pcDNA3.1-HA-nTRIP6ΔID1 and pcDNA3.1-HA-nTRIP6ΔID2 were generated by PCR amplification using nTRIP6ΔID1-VN and nTRIP6ΔID2-VN as templates, and subcloning into pcDNA3.1-HA. The mCherry-tagged, nuclear targeted blocking peptides ID1 and ID2, as well as their scrambled control versions ID1c and ID2c in pcDNA3.1 have been described^30^.

### Cell culture and transfections

C2C12 (ATCC, LGC Standards GmbH, Wesel, Germany, catalogue number CRL-1772) were cultured in Dulbecco’s modified Eagle’s medium (DMEM) supplemented with 10% foetal calf serum. Plasmid DNAs were transfected into 50% confluent cells using PromoFectin (PromoKine, Heidelberg, Germany). For luciferase assays, cells were co-transfected with the pGL3-TATA-Desmef3 reporter gene together with the β-galactosidase expression vector pcDNA3.1/Myc-His(+)/lacZ (Invitrogen GmbH, Karlsruhe, Germany) to account for possible differences in transfection efficiency. Cells were harvested 24 h post transfection and firefly luciferase activities were normalised to β-galactosidase activities within the same samples.

Synthetic siRNA duplexes were purchased from Eurofins MWG Operon (Ebersberg Germany). C2C12 cells were transfected by nucleofection with an siRNA targeting the mouse TRIP6 mRNA sequence GUCUGGAUGCUGAGAUAGA^28^ or a control siRNA targeting dsRed^60^ using the Cell Line Nucleofector^®^ Kit V (Lonza, Cologne, Germany). Cells were then seeded at low cell density to allow proliferation.

### Immunofluorescence analysis, BiFC and Laser Scanning Microscopy

Immunofluorescence analysis was performed on cells grown and transfected (when indicated) on glass coverslips coated with collagen Type I (Sigma-Aldrich), fixed for 10 min in 10% formalin and permeabilised for 10 min in 0.5% Triton X-100 in PBS. The primary antibodies were a custom-made rabbit anti-TRIP6 antibody (see below), a rat anti-HA antibody (Roche) and a mouse anti-Flag antibody (Sigma-Aldrich), and the secondary antibodies an anti-rabbit, an anti-rat and an anti-mouse Alexa Fluor 488-conjugated antibodies (Invitrogen). When indicated, nuclei were counterstained with DRAQ5 (Biostatus Ltd., Shepshed, UK). Cells were imaged by confocal microscopy (see below). The anti-TRIP6 rabbit monoclonal antibody was generated against a recombinant fragment of the mouse TRIP6 pre-LIM region corresponding to residues 104 to 282 (Epitomics Inc. Burlingame, CA). This antibody, which recognizes both TRIP6 and nTRIP6, was characterised (Supplementary Fig. S2) in comparison to a commercially available anti-TRIP6 antibody (BD Transduction Laboratories).

For Bimolecular fluorescence complementation assays (BiFC)^31^,^59^, cells were grown and transfected as described above in eight-well chamber slides (ibidi, Martinsried, Germany). Cells were imaged 24 h after transfection using a Zeiss LSM 510 Meta in confocal multitracking mode, with an x40/0.7-oil Apochromat objective (Zeiss, Jena, Germany) to generate 0.5 µm optical sections. Images were analysed using ImageJ (Rasband, W.S., ImageJ, U. S. National Institutes of Health, Bethesda, Maryland, USA, http://imagej.nih. gov/ij/1997–2011). The number of transfected cells showing Venus complementation was quantified. Linear brightness and contrast adjustments were made for illustration purposes, but only after the analysis had been made.

### Reverse transcription and real-time PCR

Total RNA was extracted from undifferentiated C2C12 cells at about 80 to 90% confluence using PeqGOLD TriFast (Peqlab Biotechnologie, Erlangen, Germany) and reverse-transcribed into cDNA. The mRNAs for *Myom2*, *Mb*, *Tnni2, Des* and the large ribosomal subunit P0 gene (*Rplp0*) used for normalisation, were quantified by real- time PCR using the ABI Prism Sequence Detection System 7000 (Applied Biosystems, Foster City, CA). The primers (Invitrogen) are described in Supplementary Table S1.

### Chromatin immunoprecipitation (ChIP)

ChIP assays were performed in undifferentiated C2C12 cells at about 80 to 90% confluence using the ChIP-IT Express kit (Active Motif, Rixensart, Belgium), following the manufacturer’s instructions. Antibodies used were a custom-made anti-TRIP6 antibody (see above), an anti-MEF2C antibody (Cell Signaling, Leiden, Netherlands) and an anti-HDAC5 antibody (Santa Cruz, Heidelberg, Germany). The isotype control antibodies were purchased from Diagenode (Liège, Belgium). For re-ChIP, complexes immunoprecipitated with the anti-TRIP6 antibody were eluted by incubation with 10 mM DTT for 30 min at 37 °C and diluted 1:500 in ChIP buffer and then re-precipitated with either the anti-MEF2C, the anti-HDAC5 or the appropriate isotype control antibody. Qualitative analysis of the occupancy and co-occupancy of the MEF2-binding regions of the *Myom2*, *Mb*, *Tnni2* and *Des* genes was performed by endpoint PCR using the primers described in Supplementary Table S2. Images of ethidium bromide-stained agarose gels were acquired using a model 1000 Super-Bright gel documentation system (PEQLAB, Erlangen, Germany). For illustration purpose, linear brightness and contrast adjustments were made on the entire pictures using ImageJ. Real-time PCR analysis was performed for the quantitative determination of the immunoprecipitated promoters upon silencing of nTRIP6. Results were calculated as percent input (ΔCt method).

### Statistical analysis

Where indicated, significant differences were assessed by t-test analysis, with values of *P* < 0.05 sufficient to reject the null hypothesis.

## Additional Information

**How to cite this article**: Kemler, D. *et al.* The LIM domain protein nTRIP6 acts as a co-repressor for the transcription factor MEF2C in myoblasts. *Sci. Rep.* **6**, 27746; doi: 10.1038/srep27746 (2016).

## Supplementary Material

Supplementary Information
