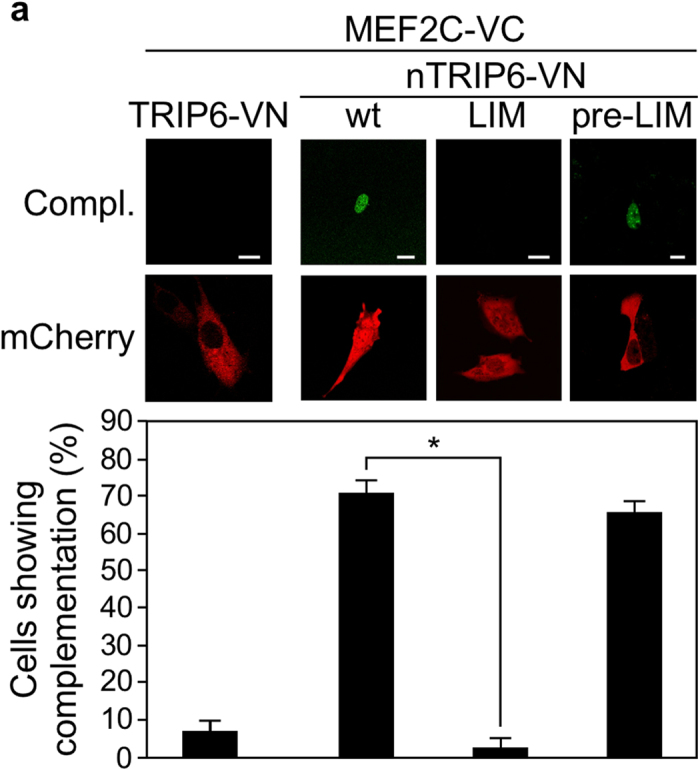# Corrigendum: The LIM domain protein nTRIP6 acts as a co-repressor for the transcription factor MEF2C in myoblasts

**DOI:** 10.1038/srep46804

**Published:** 2017-05-08

**Authors:** Denise Kemler, Oliver Dahley, Sven Roßwag, Margarethe Litfin, Olivier Kassel

Scientific Reports 6: Article number: 27746; 10.1038/srep27746 published online: 06 13 2016; updated: 05 08 2017.

This Article contains errors in Figure 2a where the pre-LIM images are a duplicate of the LIM images present in Figure 5a. The correct Figure 2a appears below as Figure 1.

## Figures and Tables

**Figure 1:**