# Supplementary material for: The LIM domain protein nTRIP6 acts as a co-repressor for the transcription factor MEF2C in myoblasts
Source: Sci Rep. 2016 Jun 13;6:27746. doi: 10.1038/srep27746 (PMC4904203; doi:10.1038/srep27746)
Supplement: Supplementary Information [file srep27746-s1.pdf]

The LIM domain protein nTRIP6 acts as a co-repressor for the transcription factor MEF2C in myoblasts

Denise Kemler, Oliver Dahley, Sven Roßwag, Margarethe Litfin, Olivier Kassel

Supplementary figures

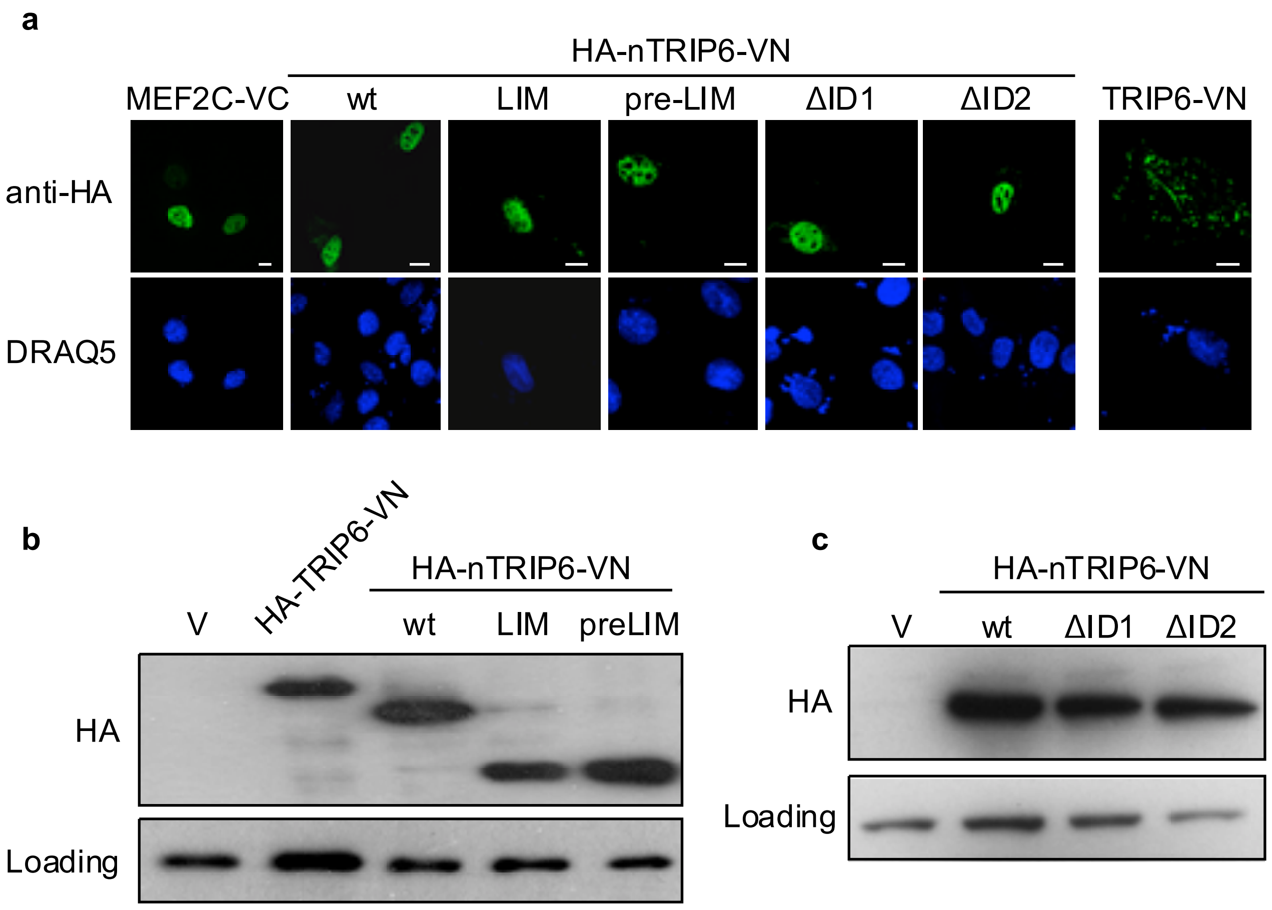

Supplementary Fig. S1. Expression of the construct used in Fig. 2. C2C12 cells were transfected with the indicated expression vectors (V, empty vector) and subjected to immunofluorescence analysis using an anti-HA antibody followed by nuclear counterstaning with DRAQ5 (a), or to Western Blotting using an anti-HA antibody and an anti-GR antibody as loading control (b,c). Scale bar in a: 10µm.

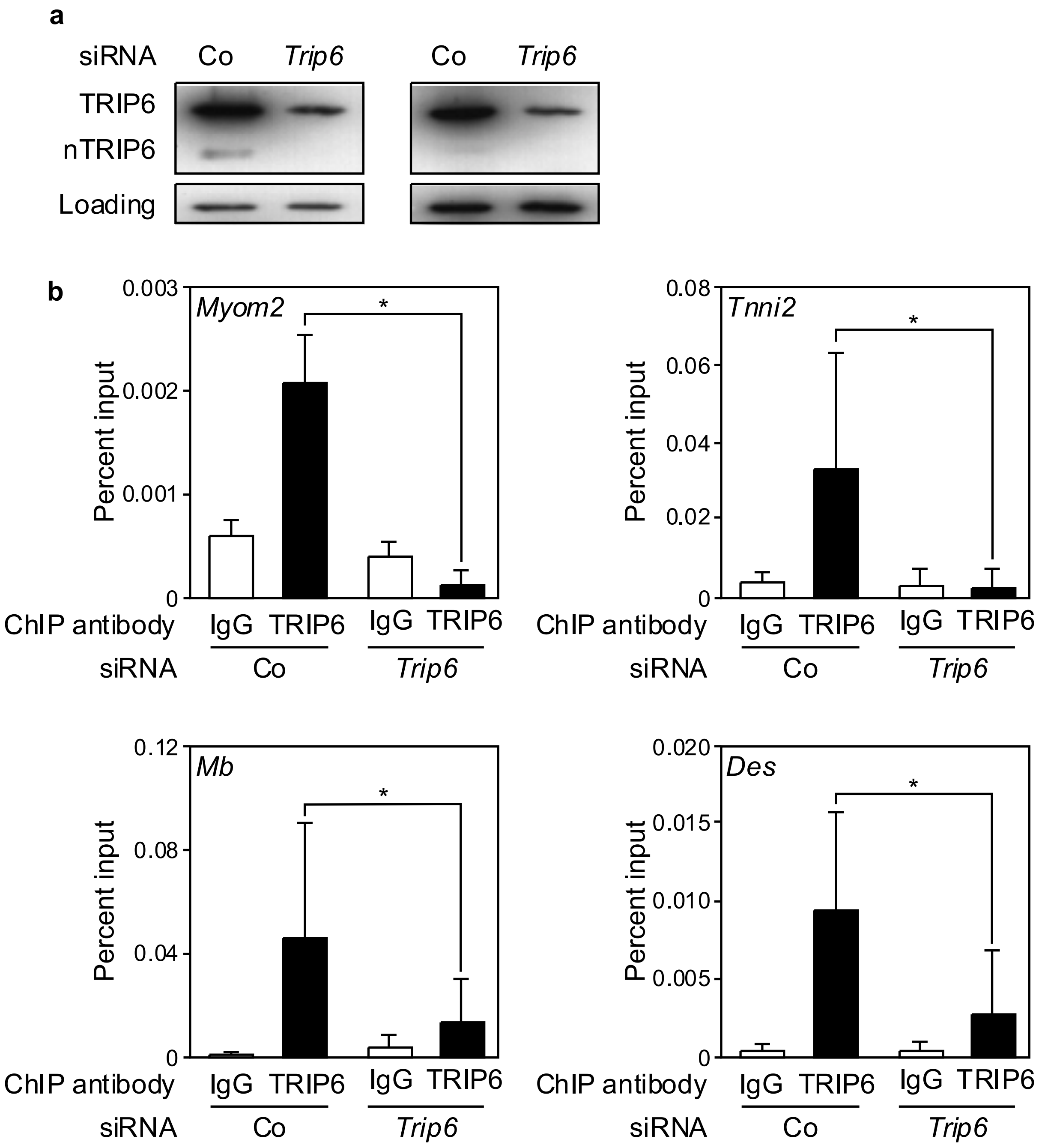

Supplementary Fig. S2. Validation of the anti-TRIP6 rabbit monoclonal antibody. C2C12 cells were transfected with either an siRNA targeting *Trip6* mRNA or a control siRNA (Co). (a) Cell lysates were subjected to Western Blotting using the anti-TRIP6 rabbit monoclonal antibody (left) or a commercially available anti-TRIP6 antibody as a control (right), and an anti-GR antibody as a loading control. (b) Chromatin immunoprecipitation (ChIP) was performed using an isotype control antibody (IgG) or the anti-TRIP6 rabbit monoclonal antibody. Enrichments of the MEF2C binding region of the indicated genes were determined by real-time PCR and are plotted as percent of input (mean ± SD of three independent experiments; *, $P < 0.05$).

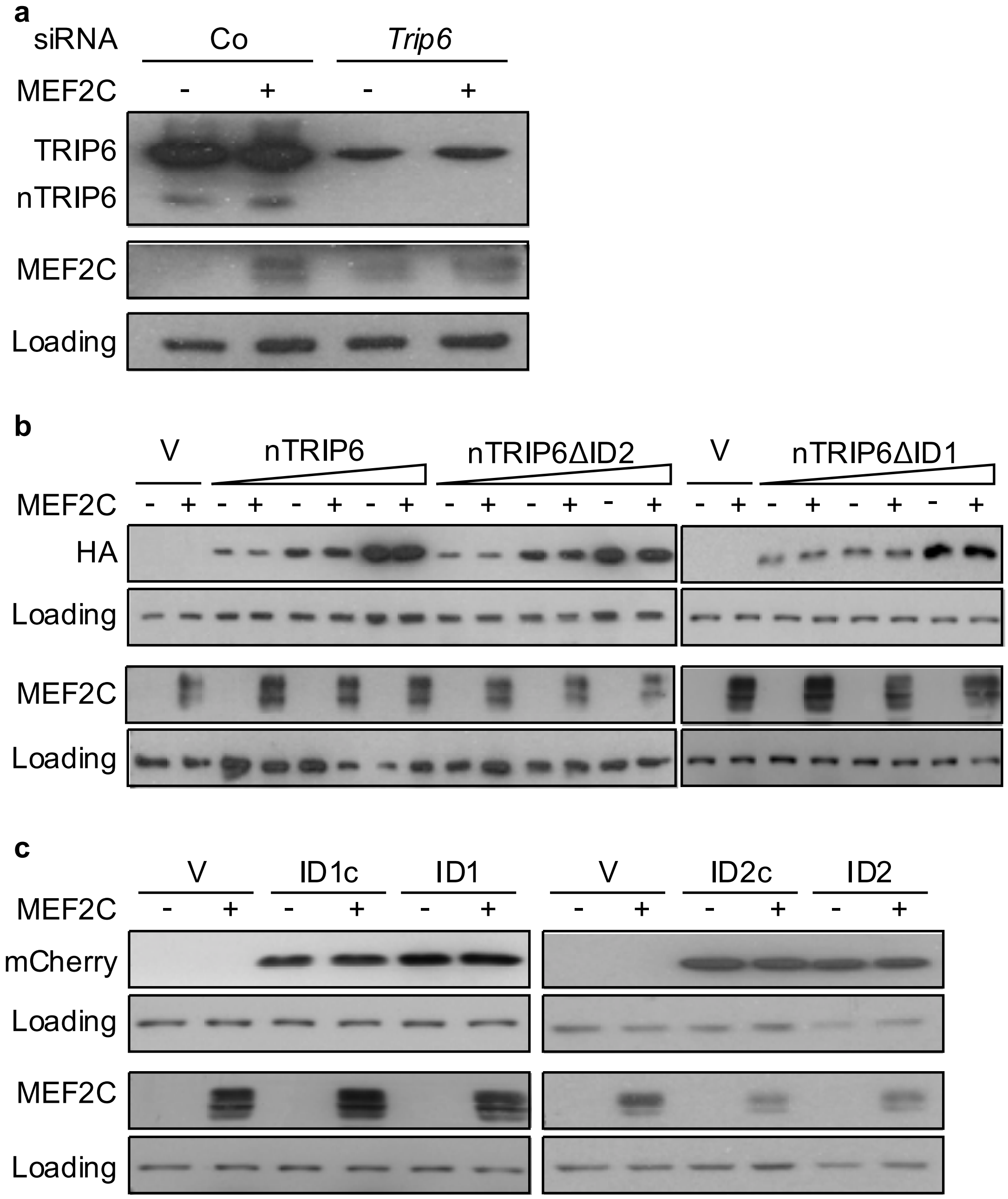

Supplementary Fig. S3. Expression controls of the experiments presented in Fig. 4. Cell lysates from the reporter gene assays presented in Fig.4a (a), Fig. 4b (b) and Fig. 4c (c) were subjected to Western Blotting using the indicated antibodies and an anti-GR antibody as a loading control.

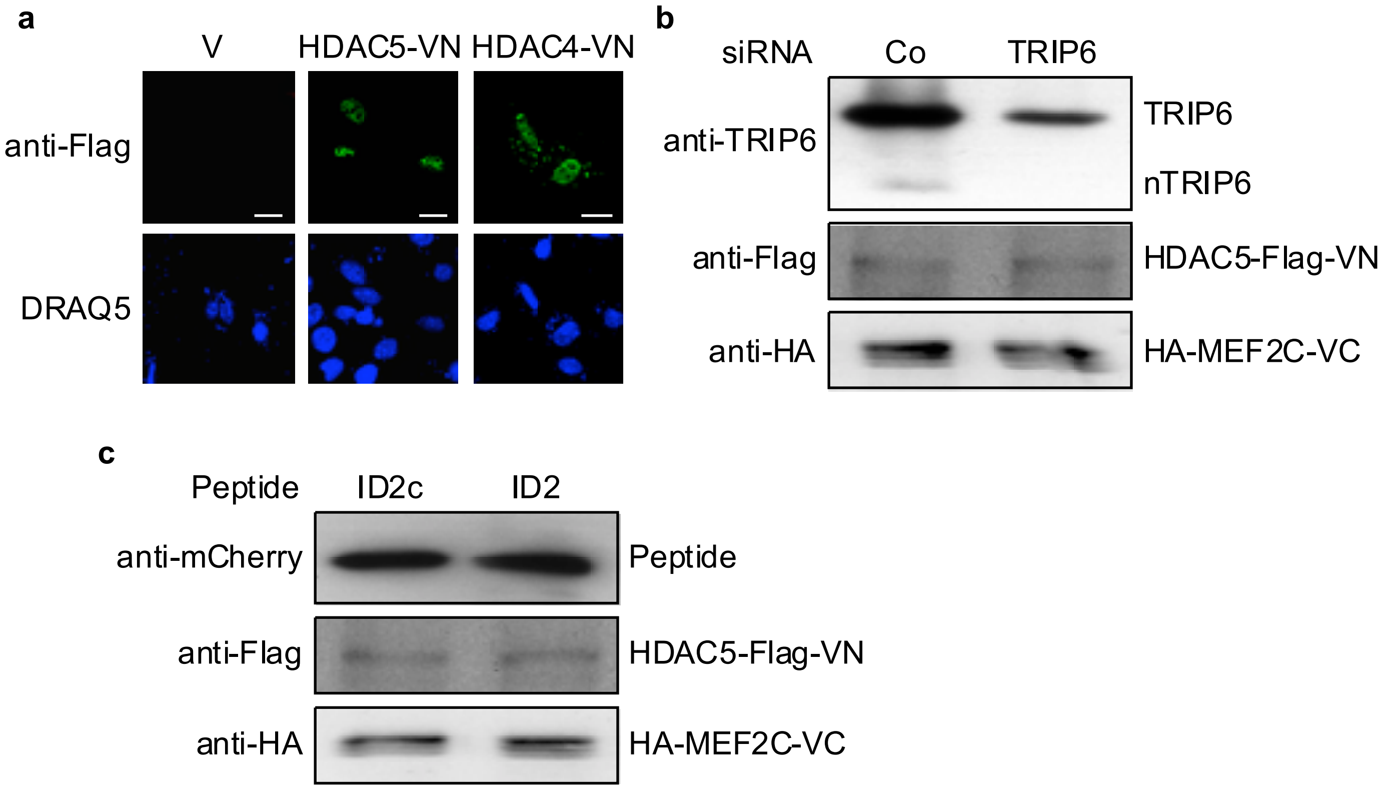

Supplementary Fig. S4. Expression controls of the experiments presented in Fig. 5 and Fig. 6. (a) C2C12 cells were transfected with either an empty vector (V) or an expression vector for either HDAC5 or HDAC4 fused to VN and subjected to immunofluorescence analysis using an anti-Flag antibody followed by nuclear counterstaning with DRAQ5. Scale bar: 20µm. (b,c) Cell lysates from the BiFC assays presented in Fig. 6a (b) and Fig. 6b (c) were subjected to Western Blotting using the indicated antibodies.

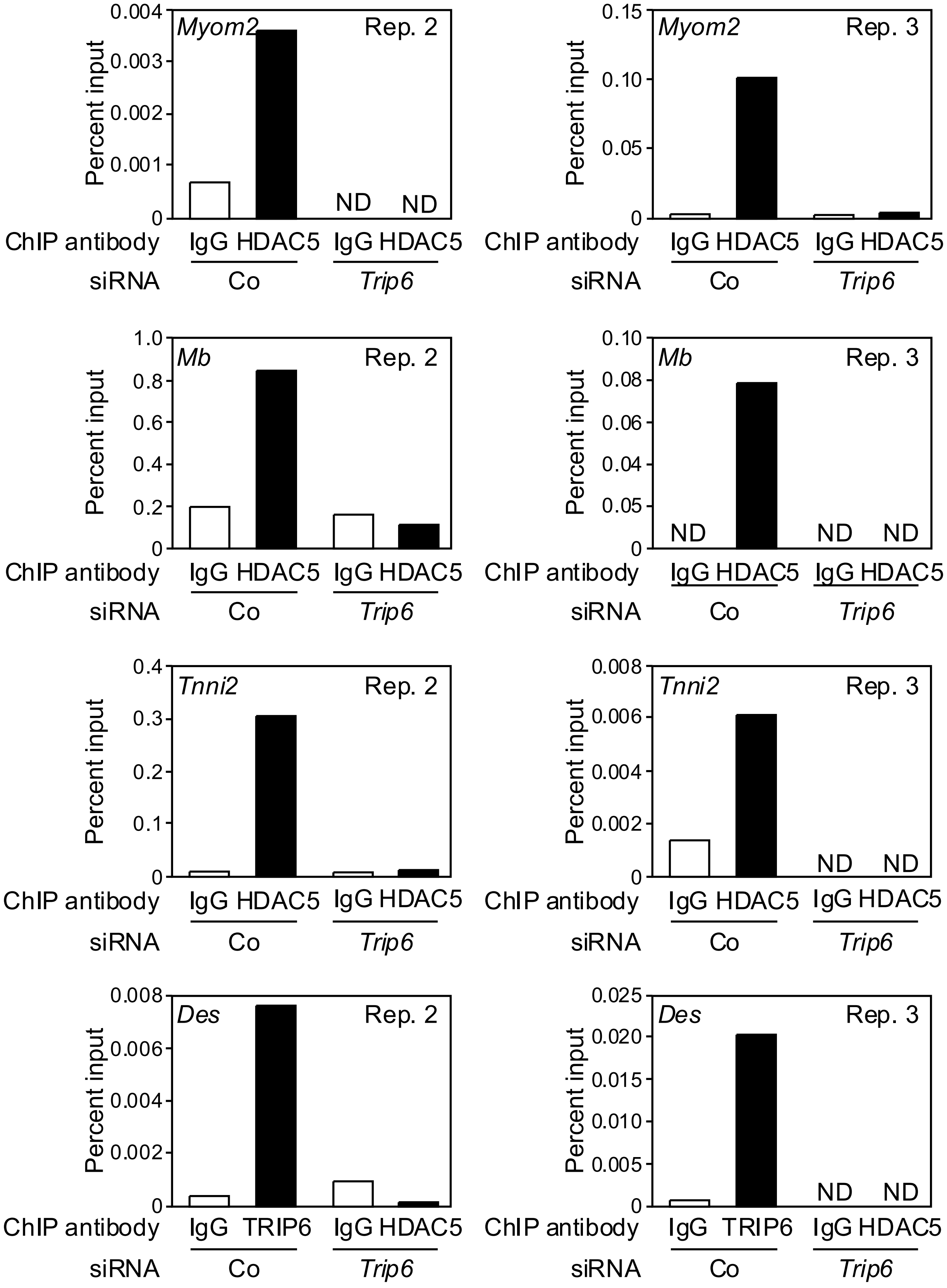

Supplementary Fig. S5. nTRIP6 mediates the recruitment of HDAC5 to MEF2C target promoters. Repetitions (Rep.) of the chromatin immunoprecipitation experiments presented in Fig. 7c. See legend of Fig. 7 for details.

Supplementary Tables

Supplementary Table S1: Real-time PCR primers

Gene name	Forward primer (5' to 3')	Reverse primer (5' to 3')
Des	CGAGCTCTACGAGGAGGAGA	GCCTCTGCAGGTCGTCTATC
Mb	AGCTGGTGCTGAATGTCTGG	AACAGACCGATGAGGACTTCC
Myom2	AAAAGACACAAGCACTTTGACC	TGGGAGGATGACTGGGTGG
Rplp0	GGACCCGAGAAGACCTCCTT	GCACATCACTCAGAATTTCAATGG
Tnni2	CATGGAGGTGAAGGTGCAGA	CTCTTGAACTTGCCCCTCAGG

Supplementary Table S2: ChIP PCR primers

Gene name	Forward primer (5' to 3')	Reverse primer (5' to 3')
Des	CCCAGAACGCCTCTCCTGTACCTT	CAGCCGTCTCCCTAGCAGCAACA
Mb	GGGCTTGTGCAAGTCCAGACAGTG	CCCTTCCTGCTACCGTGCTCAAC
Myom2	GAGCAGAGTACCCTGGGACG	TTATGGCCAGAGGAGGTGCTA
Tnni2	GCTGGCATCTTGAACTCGTC	TCAGAAAGGGCATGGAGTCTC

Supplementary Material and Methods

Western blotting

Western blot analyses were performed using a custom made anti-TRIP6 (see main Materials and Methods section), a commercially available anti-TRIP6 (BD Transduction Laboratories), an anti-MEF2C (Cell Signaling, Leiden, Netherlands), an anti-HA (clone 3F10, Roche Applied Science, Mannheim, Germany), an anti-Flag (Sigma-Aldrich, Taufkirchen, Germany), an anti-mCherry (abcam, Cambridge, UK) and an anti-GR (clone 4H2; Novocastra) which was used as a loading control.
